# Tumour stroma ratio is a potential predictor for 5-year disease-free survival in breast cancer

**DOI:** 10.1186/s12885-022-10183-5

**Published:** 2022-10-21

**Authors:** Dandan Yan, Xianli Ju, Bin Luo, Feng Guan, Huihua He, Honglin Yan, Jingping Yuan

**Affiliations:** Department of Pathology, Renmin Hospital of Wuhan University, 238 Jiefang-Road, Wuchang District, Wuhan, 430060 People’s Republic of China

**Keywords:** Breast cancer, Tumor-stroma ratio, Digital image-quantified analysis, Prognosis prediction

## Abstract

**Background:**

The tumour–stroma ratio (TSR) is identified as a promising prognostic parameter for breast cancer, but the cutoff TSR value is mostly assessed by visual assessment, which lacks objective measurement. The aims of this study were to optimize the cutoff TSR value, and evaluate its prognosis value in patients with breast cancer both as continuous and categorical variables.

**Methods:**

Major clinicopathological and follow-up data were collected for a series of patients with breast cancer. Tissue microarray images stained with cytokeratin immunohistochemistry were evaluated by automated quantitative image analysis algorithms to assess TSR. The potential cutoff point for TSR was optimized using maximally selected rank statistics. The association between TSR and 5-year disease-free survival (5-DFS) was assessed by Cox regression analysis. Kaplan–Meier analysis and log-rank test were used to assess the significance in survival analysis.

**Results:**

The optimal cut-off TSR value was 33.5%. Using this cut-off point, categorical variable analysis found that low TSR (i.e., high stroma, TSR ≤ 33.5%) predicts poor outcomes for 5-DFS (hazard ratio [HR] = 2.82, 95% confidence interval [CI] = 1.81–4.40, *P* = 0.000). When TSR was considered as a continuous parameter, results showed that increased stroma content was associated with worse 5-DFS (HR = 1.71, 95% CI = 1.34–2.18, *P* = 0.000). Similar results were also obtained in three molecular subtypes in continuous and categorical variable analyses. Moreover, in the Kaplan–Meier analysis, log-rank test showed that low TSR displayed a worse 5-DFS than high TSR (*P* = 0.000). Similar results were also obtained in patients with triple-negative breast cancer, human epidermal growth factor receptor 2 (HER2)-positive breast cancer, and luminal–HER2-negative breast cancer.

**Conclusion:**

TSR is an independent predictor for 5-DFS in breast cancer with worse survival outcomes in low TSR. The prognostic value of TSR was also observed in other three molecular subtypes.

**Supplementary Information:**

The online version contains supplementary material available at 10.1186/s12885-022-10183-5.

## Background

Breast cancer is the most common malignant tumor in women globally with an estimated 2.3 million new cases and more than 685,000 deaths worldwide in 2020 [1]. Although certain improvements have been achieved in personalizing therapy, the regular prognostic and predictive parameters currently used to stratify disease risks have remained largely unchanged for nearly two decades. Optimizing risk stratification by searching more prognostic factors to prevent overtreatment or undertreatment is therefore essential.

The tumor microenvironment, also known as tumor-associated stroma, refers to a complex mixture of non-tumor cells, including fibroblasts, immune cells, pericytes, and endothelial cells, which actively participate in the growth and progression of solid tumors [2]. Evidence suggested that tumorigenesis is controlled by the complex interactions between stromal elements and cancer cells, and many in vivo and in vitro studies have shown that tumor stroma plays a tumor-promoting role [3–5]. Tumor stroma promotes tumor progression and metastasis by producing various nutrients, chemokines, growth factors, and cytokines [6]. In recent years, the tumor microenvironment has been actively incorporated in the search for new prognostic and predictive biomarkers. Tumor–stroma ratio (TSR) refers to the ratio of tumor stroma to cancer cells within the tumor tissue. TSR is a promising prognostic factor to assess the tumor microenvironment. Previous studies have explored the prognostic value of TSR in a range of invasive solid tumors, including primary colorectal carcinomas [7], epithelial ovarian cancer [8], gastric cancer [9], and breast cancer [10]. A majority of these previous studies demonstrated poor prognostic outcomes in patients with stroma-high tumors.

As a heterogeneous disease, breast cancer comprises multiple distinct subtypes that differ genetically, pathologically, and clinically. The heterogeneity among patients with breast cancer has attracted enormous interest in predictive and prognosis biomarkers, as these factors could effectively improve clinical decision-making in early-stage breast cancer. Previous studies have assessed the association between TSR and survival outcomes in patients with breast cancer; most of them suggested that patients with stroma-high tumor showed a relatively poor prognosis than patients with stroma-low tumor [10–13]. However, in luminal tumors, a recent study found that low TSR was associated with a favorable outcome [11]. Moreover, most of these previous studies employed a visual assessment approach using a predefined cutoff point of 50% stroma to divide patients into stroma-high or stroma-low group to assess TSR in whole tumor sections. Few studies have used automated quantitative image analysis algorithms to assess TSR, except for one recent study [11].

Considering the existence of a subjective element in visual assessment methods for TSR and inter-investigator variability, we employed a digital image-quantified analysis to calculate TSR. The aims of this study were to use a digital image-quantified analysis algorithm to assess TSR and evaluate its clinical significance as categorical and continuous variables in survival outcomes among different breast cancer subtypes.

## Methods

### Study population

The patients in the present study were selected from a clinical database consisting of patients with invasive breast cancer who were primarily treated with surgery at Renmin Hospital of Wuhan University between 2002 and 2007. Major clinicopathologic characteristics, including age, menopausal status, histopathological grade, nodal status, estrogen receptor (ER) status, progesterone receptor (PR) status, human epidermal growth factor receptor-2 (HER-2) status, and tumor size, were obtained from pathological reports. Inclusion criteria were patients with follow-up data and hematoxylin and eosin (H&E) slides for primary breast tumors. Exclusion criteria were a history of cancer or the absence of major clinicopathologic characteristics and resected tissue slides. A total of 240 patients were included for analysis. This retrospective study was approved by the Ethics Committee of the Renmin Hospital of Wuhan University (approval no. WDRY2021-k153, date: 2021–09-26). Informed consent was waived by the Ethics Committee of the Renmin Hospital of Wuhan University because of the retrospective nature of the study.

### Tissue microarray (TMA) construction

TMA was constructed by a standard procedure in collaboration with Shanghai Aodo Biotechnology Co., Ltd. (Shanghai, China). After all donor tumors were examined on H&E slides, the tumor block representing the deepest tumor infiltration into the wall (i.e., the most invasive part of the primary tumor) was marked by an experienced breast pathologist. Then, the corresponding areas in the paraffin block were marked. Two 3 × 1 mm^2^ cores were sampled from each donor tumor using punched cores and placed within the TMA block to ensure the reproducibility and homogenous staining of the slides. In this study, each TMA block contained 70 cylinders, and seven TMAs with 480 cores were constructed.

### Immunohistochemical (IHC) staining

Cytokeratin (CK) IHC staining was used to distinguish tumor cells from stromal cells in the TMA. Paraffin sections with 4 µm thickness were cut, deparaffinized, and dehydrated according to the standard procedures. For antigen retrieval, sections were incubated with 0.01 M citrate buffer (pH 6.0) and microwaved at 90 °C for 20 min. Next, the sections were blocked with 10% normal goat serum at 37 °C for 20 min to reduce background intensity. Subsequently, the sections were incubated with mouse anti-human CK monoclonal antibody (species: mouse; dilution 1:100; ZSGB-BIO, Beijing, China) overnight at 4 °C. The slides were washed in TBS, treated with horseradish peroxidase-labeled anti-mouse antibodies (species: goat) for 20 min at room temperature and dripped with 3,3-diaminobenzidine (dilution: 1:500; DAKO, Denmark). The sections were counterstained with hematoxylin, sealed with resin mount, and digitally scanned at × 20 magnification using a KF-PRO-005 scanner (KFBIO company, Ningbo City, China).

### TSR assessment

Digital image processing was preformed using the open-source computer vision library, OpenCV (https://opencv.org/), which provides many general algorithms for computer vision process and machine learning. A morphology-based pathological image segmentation algorithm was used to segment the tumor epithelium and stroma. Image pre-processing was applied by Gaussian filter to reduce the effect of noise. Edge detection was performed to find out the contours of the tissue area. The area outside the outline was regarded as the background and excluded from analysis. A distinct difference in hue was found between the tumor (brown) and stroma (off-white). According to the rule of color space distribution, all images were converted from red–green–blue to hue–saturation–value though color space transformation, and the hue channel was selected to generate gray-scale images. Otsu method (maximal variance between-class) was implemented to find the optimal threshold for image binarization automatically. The localized holes and discrete spots caused by tissue distribution can be overcome by repairing holes with dilation operations and removing speckles with erosion operations. Lastly, the region with a pixel values of 255 and 0 represent the tumor and stromal region, respectively. Data output by OpenCV provided the area and percentage values of tumor epithelium and stroma in each TMA core. The one with the highest proportion of matrix between the two TMA cores was recorded as the final result. TSR is defined as area _tumor_ /(area _tumor_ + area _stroma_) × 100%.

### Statistical analysis

IBM SPSS statistics (version 23.0) and R (version 4.2.0) were used to perform statistical analyses. The endpoint of interest in this study was disease-free survival (DFS), which is defined as the total survival time from the date of surgery to the local, regional, or distant recurrence of breast cancer. Patients who died without a recurrence or were lost to follow-up were censored. Breast cancer subtypes were classified into human epidermal growth factor receptor 2 (HER2)-positive breast cancer, triple-negative breast cancer (TNBC), and luminal-HER2-negative breast cancer based on IHC (for ER and PR) and in situ hybridization (HER2 2 +). The hormone receptor-positive (luminal) subtype was not divided into luminal A or luminal B-like tumors because of the limit of sample size.

For categorical variable analysis, the maximally selected rank statistics [14] was used to identify the potential TSR cutoff point. This point was chosen to divide the patients into stroma-low and stroma-high tumors associated with differences in 5-year disease-free survival (5-DFS). The correlations between TSR categories and major clinicopathologic characteristics were assessed by χ^2^ test. Univariate and multivariate analyses of DFS were assessed by Cox regression model. The hazard ratios (HRs) for continuous TSR variable units for 5-DFS were reported for units of 10%. The 95% confidence intervals (CIs) and HRs for Cox regression were calculated using the built-in function of the SPSS software. In multivariate regression analysis models, the covariates were age (≤ 50 vs > 50), tumor size (T1 vs T2 vs T3), nodal status (positive vs negative), histopathological grade (I vs II vs III), ER status (positive vs negative), PR status (positive vs negative), HER-2 status (positive vs negative), and menopausal status (premenopausal vs postmenopausal). Kaplan–Meier analysis and log-rank test were used to assess the significance in survival analysis. All *P* values were two sided, and a *P*-value less than 0.05 was considered statistically significant in all comparisons.

## Results

### Patients characteristics

Approximately 462 patients with invasive breast cancer were identified. Among the 462 patients, 222 patients were excluded for the following reasons: a history of cancer, recurrent or metastatic breast cancer, absence of major clinicopathologic characteristics or resected tissue slides, and absence of follow-up data. In total, 240 female patients with breast cancer were included in the present analysis. Among the 240 cases, 63 (26.3%) were TNBC, 61 (25.4%) were HER2-positive breast cancer, and 116 (48.3%) were luminal-HER2-negative tumors. The median age at the time of diagnosis was 49 years (age range 29–78 years). All patients were followed up for 60 months, the median follow-up period was 46 months (range 2–60 months), and the 5-DFS rate was 62.0%. The median survival period for TNBC and HER2-positive breast cancer were 51 and 56 months, respectively. The median survival period for luminal–HER2- negative breast cancer was not reached.

### TSR evaluation

**Fig. 1:**
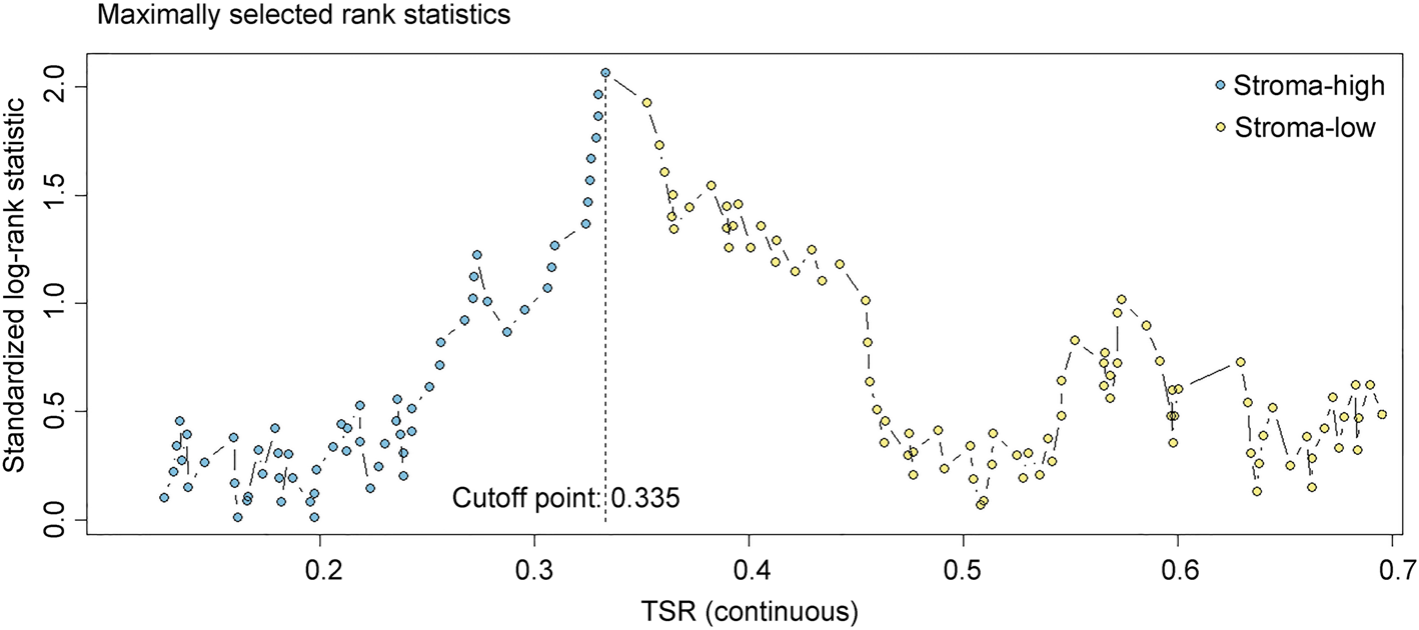
The cutoff point determination analysis. The optimal cutoff point to divide TSR as stroma-low and stroma-high were determined by maximally elected rank statistics method

**Fig. 2:**
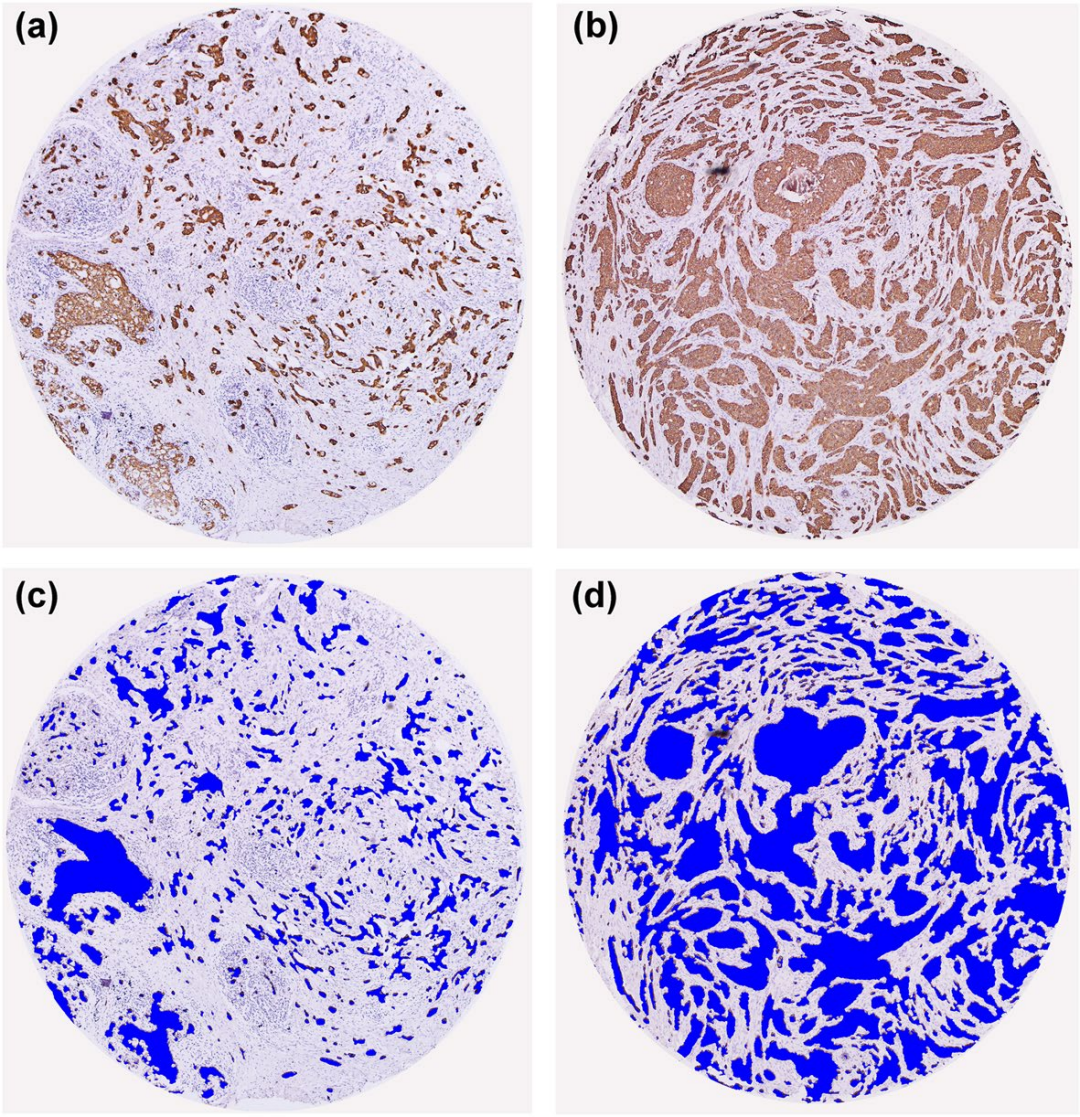
Representative IHC staining image and the corresponding segmented image of tumor epithelium (blue) using the pixel classifier algorithm in OpenCV. **A**, **C** The examples of low TSR (i.e., stroma-high, × 100) and **B**, **D** high TSR (i.e., stroma-low, × 100) tumor

Patients were divided into stroma-low (TSR > 33.5%) and stroma-high (TSR ≤ 33.5%) groups according to the optimal cutoff point determined for the total cohort (Fig. 1). Based on the cutoff point, 153 (63.75%) patients were determined to be stroma-low (high TSR) cases and 87 (36.25%) patients were determined to be stroma-high (low TSR) cases. Representative examples of high and low TSR images produced by the pixel classifier in OpenCV are shown in Fig. 2.

### Correlation between TSR and clinical pathological parameters

The correlation between TSR and clinical pathological parameters is shown in Table 1. Among the included clinical pathological parameters, TSR was significantly correlated with ER status (*P* = 0.005) and PR status (*P* = 0.035), and tumor size had marginally statistical difference between the low and high TSR groups (*P* = 0.051). Other clinical pathological parameters, including age (*P* = 0.578), menopausal status (*P* = 0.075), histopathological grade (*P* = 0.512), molecular subtypes (*P* = 0.91), nodal status (*P* = 0.079), and HER-2 status (*P* = 0.132) did not show statistical differences between the low and high TSR groups.

**Table 1:** Baseline parameters and distribution of TSR in clinico-pathological subgroups

Characteristics	Number of patients	Stroma-low		Stroma-high		χ^2^ value	*P* value
		N	%	N	%		
Age							
≤ 50	149	97	63.4%	52	59.8%	0.310	0.578
> 50	91	56	36.6%	35	40.2%		
Histopathological Grade							
I	40	27	17.6%	13	14.9%	1.338	0.512
II	141	92	60.1%	49	56.3%		
III	59	34	22.2%	25	28.7%		
Molecular subtypes							
TNBC	63	37	24.2%	26	29.9%	4.799	0.091
HER-2 positive	61	34	22.2%	27	31.0%		
Luminal-HER2-negative	116	82	53.6%	34	39.1%		
Nodal status							
Positive	131	77	50.3%	54	62.1%	3.085	0.079
Negative	109	76	49.7%	33	37.9%		
ER status							
Positive	106	78	51.0%	28	32.2%	7.946	0.005
Negative	134	75	49.0%	59	67.8%		
HER-2 status							
Positive	61	34	22.2%	27	31.0%	2.272	0.132
Negative	179	119	77.8%	60	69.0%		
PR status							
Positive	107	76	49.7%	31	35.6%	4.426	0.035
Negative	133	77	50.3%	56	64.4%		
Menopausal status							
Premenopausal	134	92	60.1%	42	48.3%	3.161	0.075
Postmenopausal	106	61	39.9%	45	51.7%		
Tumor size (in cm)							
T1 (T ≤ 2)	35	21	13.7%	14	16.1%	5.945	0.051
T2 (2 < T ≤ 5)	162	111	72.5%	51	58.6%		
T3 (T > 5)	43	21	13.7%	22	25.3%		

### Prognostic value of TSR as a continuous variable

**Fig. 3:**
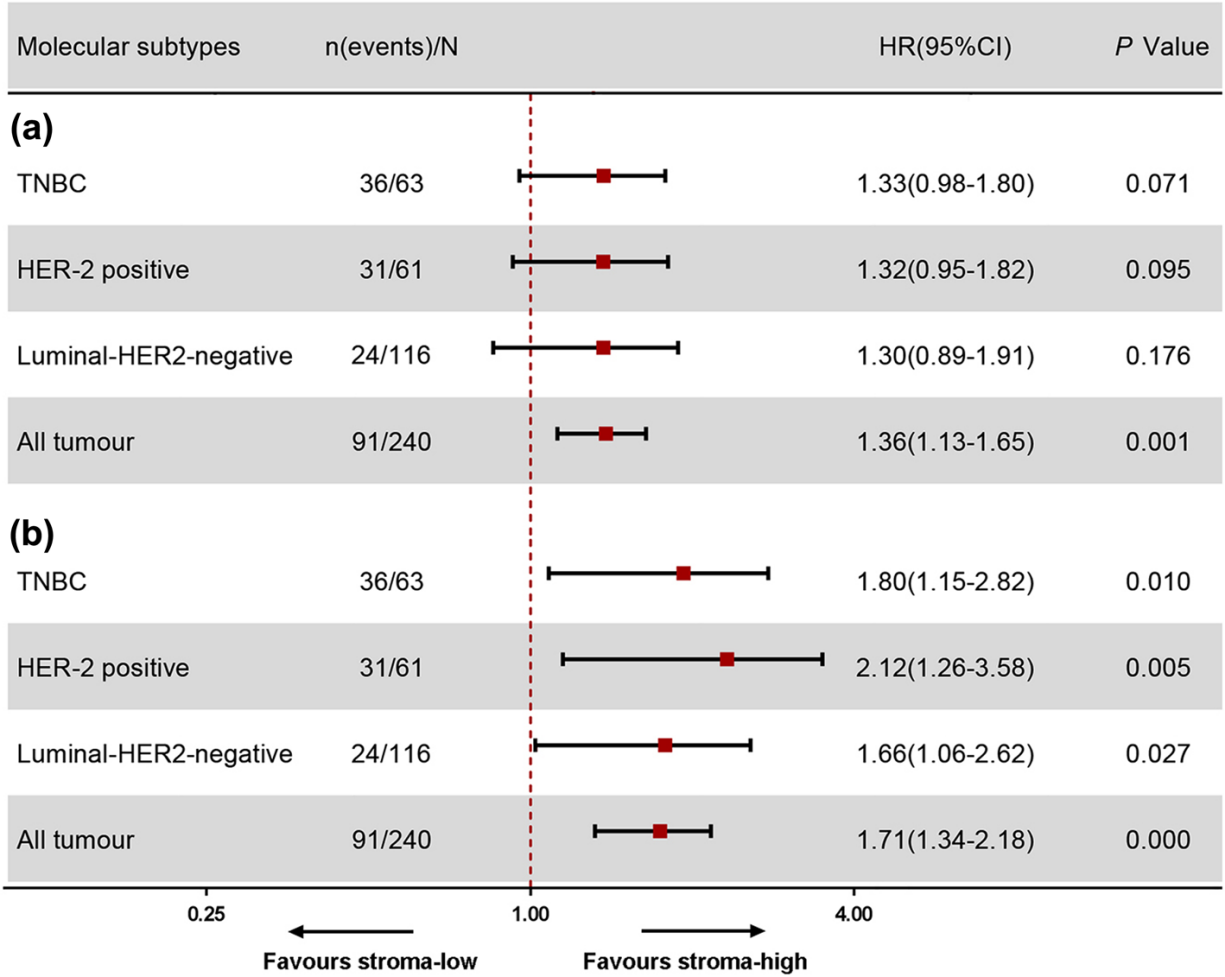
The prognostic value of TSR as a continuous variable. *P* values have been obtained from Cox-regression analysis. **A** Disease-free survival by univariate analysis (**B**) and multivariate analysis including all baseline parameters; TNBC = triple-negative breast cancer

The prognostic value of TSR as a continuous variable for 5-DFS was assessed by Cox proportional hazards in all of the breast cancer series. In univariate analysis, TSR was not significantly associated with 5-DFS in TNBC (HR = 1.33, 95% CI = 0.98–1.80, *P* = 0.071), HER2-positive breast cancer (HR = 1.32, 95% CI = 0.95–1.82, *P* = 0.095), or luminal– HER2-negative breast cancer (HR = 1.30, 95% CI = 0.89–1.91, *P* = 0.176; Fig. 3A). By contrast, in the total breast cancer series, patients with increased tumor-stromal content displayed a significantly shorter DFS compared with patients with lower tumor-stromal content (HR = 1.36, 95% CI = 1.13–1.65, *P* = 0.001; Fig. 3A).

In the multivariable analysis, patients with increased tumor-stromal content in the TNBC group showed significantly shorter DFS than patients with lower tumor-stromal content (HR = 1.80, 95% CI = 1.15–2.82, *P* = 0.010; Fig. 3B). Similarly, increased tumor-stromal content in patients with HER2-positive breast cancer resulted in a markedly shorter DFS than lower tumor-stromal content (HR = 2.12, 95% CI = 1.26–3.58, *P* = 0.005; Fig. 3B). Tumor-stromal content was also significantly associated with a shorter DFS in patients with luminal–HER2-negative breast cancer (HR = 1.66, 95% CI = 1.06–2.62, *P* = 0.027; Fig. 3B). In addition, in the total breast cancer series, patients with increased tumor-stromal content displayed a significantly shorter DFS compared with patients with lower tumor-stromal content (HR = 1.71, 95% CI = 1.34–2.18, *P* = 0.000; Fig. 3B).

### The prognostic value of TSR as a categorical variable

When TSR was assessed as a categorical variable (Table 2), the Cox proportional hazards model demonstrated that low TSR (i.e., stroma-high) predicts poor outcomes for 5-DFS in univariate (HR = 2.69, 95% CI = 1.78–4.06, *P* = 0.000) and multivariate analyses (HR = 2.82, 95% CI = 1.81–4.40, *P* = 0.000). Similarly, low TSR was also associated with poor outcomes for 5-DFS both in univariate and multivariate analyses for HER2-positive breast cancer and TNBC (Supplementary Tables 1 and 2). By contrast, in luminal–HER2-negative breast cancer, 5-DFS did not show significant differences between the low TSR and high TSR groups in the univariate analysis model. However, in multivariate analysis, 5-DFS showed a remarkable difference in favor of stroma-low tumors after adjusted for confounders (Supplementary Table 3). These results suggested that TSR is an independent prognostic factor for breast cancer 5-DFS in the model independent of age, lymph node status, histopathological grade, ER status, PR status, HER-2 status, menopausal status, and tumor size.

**Table 2:** Univariate and multivariate analysis of the Breast Cancer disease-free survival by Cox regression analysis

Characteristics	Number of patients	Univariable analysis		Multivariable analysis	
		HR(95%CI)	*P* value	HR(95%CI)	*P* value
Age					
	240	0.99(0.97–1.01)	0.369	0.98(0.95–1.02)	0.394
Histopathological Grade					
I	40	ref		ref	
II	141	3.21(1.15–8.98)	0.026	1.69(0.59–4.87)	0.327
III	59	15.28(5.48–42.59)	0.000	5.92(1.98–17.74)	0.001
Nodal status					
Negative	109	ref		ref	
Positive	131	5.04(2.97–8.54)	0.000	3.78(2.17–6.60)	0.000
ER status					
Negative	134	ref		ref	
Positive	106	0.56(0.38–0.83)	0.004	0.95(0.63–1.43)	0.796
HER-2 status					
Negative	189	ref		ref	
Positive	51	1.95(1.26–3.01)	0.003	1.78(1.09–2.89)	0.020
PR status					
Negative	133	ref		ref	
Positive	107	0.66(0.50–0.88)	0.005	1.00(0.72–1.40)	0.986
Menopausal status					
Premenopausal	134	ref		ref	
Postmenopausal	106	1.41(0.934–2.12)	0.103	1.45(0.76–2.79)	0.261
Tumor size (in cm)					
T1 (T ≤ 2)	35	ref		ref	
T2 (2 < T ≤ 5)	162	3.62(1.31–9.95)	0.013	3.23(1.16–8.99)	0.025
T3 (T > 5)	43	8.31(2.90–23.79)	0.000	4.26(1.45–12.53)	0.009
TSR					
Stroma-low	153	ref		ref	
Stroma-high	87	2.69(1.78–4.06)	0.000	2.82(1.81–4.40)	0.000

**Fig. 4:**
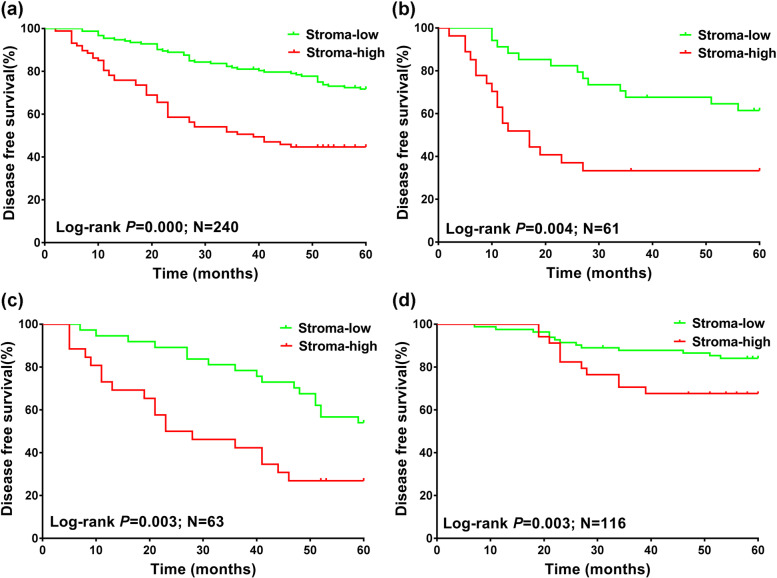
Kaplan–Meier analyses for prognosis of breast cancer patients with stroma-high and stroma-low in different molecular subtypes. **A** Disease-free survival in all type of breast cancer. **B** Disease-free survival in HER2-positive breast cancer. **C** Disease-free survival in TNBC. **D** Disease-free survival in luminal–HER2-negative breast cancer. *P* values were derived from log-rank test; TNBC = triple- negative breast cancer. HR = hazard ratio

Next, the total breast cancer series and the three subgroups, including HER2-positive breast cancer, TNBC, and luminal-HER2-negative breast cancer, were included in the Kaplan–Meier analyses. Log-rank test showed that patients with stroma-high tumor (low TSR) displayed a worse survival in the total breast cancer series (*P* = 0.000, Fig. 4A). Similarly, patients with stroma-high tumor (low TSR) also displayed a worse prognosis in HER2-positive breast cancer, TNBC, and luminal-HER2-negative breast cancer (all *P* < 0.05, Fig. 4B-D).

## Discussion

Growing evidence highlights the importance of tumor microenvironment in tumor progression [15–17]. The tumor microenvironment, especially the tumor stroma, has been recognized as an important driver of tumor progression [18]. However, indicators of tumor stroma have not yet been integrated into routine clinical decision-making. A novel parameter reflecting the content of tumor-associated stroma is TSR, which has been extensively described as a rich source of prognostic information for various solid cancer types. The biological role of TSR is recorded in different cancer types [19–21]. Previous research has focused on the prognostic effect of TSR, as a high proportion of tumor stroma is often associated with poor prognosis [22, 23]. However, the results were not always consistent in different molecular subtypes.

In the present study, the prognostic value of TSR was assessed in 240 patients with breast cancer, and the clinically relevant subgroups for breast cancer prognosis were analyzed. The main findings of this study are as follows. First, patients with a stroma-high tumors (low TSR) had worse survival outcomes compared with patients with stroma-low tumors (high TSR). Second, the prognostic value of TSR was not affected by age at onset, histopathological grade, lymph node status, ER status, PR status, HER2 status, menopausal status, or tumor size. Third, subgroup analysis according to the molecular subtypes suggested that the prognostic significance of TSR in the HER2-positive breast cancer, TNBC, and luminal-HER2-negative breast cancer subgroups did not differ from the prognostic value in the total breast cancer series. Fourth, TSR was a continuous variable. Categorical and continuous variable analyses were performed on TSR, and both analyses gave similar results on the performance of TSR for prognosis prediction.

The prognostic value of TSR in breast cancer has been reported in several studies. TSR was first identified as a prognostic factor in breast cancer by Kruijf et al, who demonstrated that patients with stroma-rich tumors, especially TNBC, have a higher risk of recurrence [24]. Dekker et al. assessed the prognostic value of TSR in premenopausal patients with breast cancer with node-negative status, the results indicated that TSR is an independent prognostic parameter for DFS and is independently associated with locoregional recurrence; this finding validated the prognostic value of TSR in breast cancer [25]. The prognostic value of TSR was also confirmed in ER-positive breast cancer [26] and inflammatory breast cancer [27]. In line with these previous studies, our research revealed that TSR was positively associated with 5-DFS, and patients with stroma-high tumors (low TSR) have worse survival outcomes than patients with stroma-low tumors (high TSR).When TSR was assessed as a categorical variable, univariate and multivariate analyses showed that TSR was associated with 5-DFS. When TSR was analyzed as a continuous variable, univariate and multivariate analyses revealed that TSR was associated with 5-DFS in the total breast cancer series. However, in the subgroup analysis, TSR was not statistically significant in all of the three subtypes in the univariate analyses but was significant in the multivariate analysis. The different outcomes could be because some clinical pathological parameters were not considered in the univariate analysis. The meaningless values may be caused by confounding factors [28]. Multivariate analysis considers the influence of multiple factors and excludes confounding factors, resulting in different results. Moreover, multivariable analysis showed that histopathological grade, nodal status, tumor size, HER-2 status, and TSR were independent prognostic factors of breast cancer.

According to the recommended TSR evaluation method, the tissue section with the most invasive part of the primary tumor should be selected to evaluate TSR [24, 29]. TMAs can quantify features and emphasize the extent of the tumor-stroma interface, it is also suitable for large sample detection, computer recognition, and automatic analysis [30]. In the process of TMA construction, the tumor block representing the deepest tumor infiltration into the wall was selected, and two cores were sampled from each donor tumor to ensure the reproducibility and homogenous staining of the slides. These conditions can make the TSR evaluation results more objective and accurate.

A reliable tumor area assessment method is the basis for exploring the prognostic value of TSR. Visual inspection by experienced pathologists [31] and computer assessment analysis [11] are the two main methods for assessing TSR. Currently, TSR is largely assessed in the H&E staining section, which may not accurately identify the boundary of tumor nests because of the low contrast between tumor and stroma in some regions. These factors may affect the reproducibility of results and make accurate identification and analysis difficult. CK IHC staining can label tumor cells and can be used to distinguish tumor cells from stromal cells [32]. Therefore, the IHC staining of CK was used to specifically label tumor cells in this study, and the results showed a strong color contrast, in which tumor cells were marked in brown and tumor stroma were marked in off-white. Digital image quantification analysis was applied to TSR assessment based on the IHC staining of CK. In this study, the optimal cut-off TSR value was 0.335, which corresponds to approximately 66.5% of stroma and 33.5% of tumor. The stromal value cut-point of 66.5% was slightly higher than the predefined cut-point for 50% stroma in other studies. These results are similar to a previous study, which found that in colorectal cancer, the cutoff point of stroma value based on convolutional neural networks is higher than the 50% stroma visual assessment [33]. The differences in cutoff points between visual assessment and computer assessment suggested that there may be a common discrepancy between humans and computers when evaluating tumor pathology images.

In our study, the optimal cut-off TSR value determined by maximally selected rank statistics, which was used to distinguish the stroma-low and stroma-high groups. However, it should be emphasized that the proportion of tumor stromal content is a continuous variable, which can reach any proportion from 0% to nearly 100%. This suggests that TSR is a continuous parameter of tumor cell interaction with stromal components rather than a marker of a specific tumor subtype. In other words, TSR could be viewed as an indirect measure of the stroma’s contribution to malignant progression, which might be similar to the proliferation marker Ki67 to some extent. The results of the continuous variable analysis of TSR were consistent with a previous study, showing that in endometrial carcinoma, TSR is associated with poorer survival outcomes when used as a continuous variable [34]. The continuous analysis of tumor stromal content is more relevant from a statistical point of view and provides a more accurate description of tumor biological behavior. In future research, if TSR can be automatically quantified from H&E sections, it will give a more accurate description of tumor biology, which will lead to more accurate individualized treatment and prognosis prediction for patients. Nonetheless, dividing TSR into stroma-high and stroma-low groups might be more practical for future clinical applications, because clinical trials are easier to implement in groups of patients based on categorical variables. Therefore, in the present study, continuous and categorical variables were analysed, and both analyses gave similar prognostic prediction results.

Another important question is how the tumor stroma contributes to the prognosis of breast cancer. It is known that TSR is composed of fibroblasts, immune cells, endothelial cells, and other supporting cells. These cells could be recruited by cancer cells from nearby endogenous host stroma, which could in turn promote tumor angiogenesis, proliferation, metastasis, and invasion [35]. Previous studies have identified events that occur in the stromal compartment during carcinogenesis, including fibroblast recruitment, stroma remodeling, immune cells migration, and angiogenesis, which may influence tumor progression [36, 37]. To date, studies on the effect of tumor-associated stroma on epithelial tumor progression have largely focused on functional in vitro studies. Cancer-associated fibroblasts is one of the factors critically involved in cancer progression. They regulate the biological function of stromal and tumor cells via intercellular contact, synthesize and remodel the extracellular matrix, elevate the proliferation rate, release numerous cytokines (such as vascular endothelial growth factor and stromal cell-derived factor 1) that lead to angiogenesis, and thus promote cancer initiation and development [37, 38]. These tumor-associated stromal cells also secrete a number of pro-tumorigenic factors, such as stromal-derived factor-1α, IL-6, IL-8, vascular endothelial growth factor, matrix metalloproteinases, and tenascin-C. These factors recruit additional tumor and pro-tumorigenic cells into the developing microenvironment, which may in turn contribute to tumor progression [38].

However, several limitations should be recognized in the present study. First, the present cohort was retrospectively assembled. The number of patients included in this study was relatively small, especially the number of patients with TNBC and HER2-positive breast cancer. Second, a period of 10 or 15 years is commonly used in breast cancer research. The follow-up period of this study was not long enough owing to the lack of available follow-up data. Previous study found that the high-risk period of breast cancer recurrence is 1–2 years after surgery, and the risk of recurrence decreases rapidly within 2–5 years and returns to a stable period within 5–12 years [39]. These findings indicated that 5-DFS might reflect the primary endpoint to some extent. Third, although the most invasive parts of the primary tumors were selected to construct the TMAs, it might not be representative for the amount of stroma in the entire resected tissue specimen, as different sampling approaches would remarkably impact TSR. Future whole-slide H&E-stained image analysis could reduce or eliminate variations in the TSR assessment results. Therefore, a large, updated retrospective study, which takes into account an appropriate follow-up period, and/or a prospective cohort study with TSR values evaluated on whole-slide H&E-stained images should be implemented to validate the prognostic value of TSR in the next clinical implementation.

## Conclusion

In summary, the results showed that digital image-quantified analysis estimation of TSR using TMA validates the findings that low TSR is an independent factor for poor prognosis in breast cancer. Moreover, the prognosis value of TSR was not affected by age at onset, histopathological grade, lymph node status, ER status, PR status, HER2 status, menopausal status, or tumor size. Further large prospective cohort studies combined with molecular biological mechanism studies should be implemented to validate the prognostic value of TSR in the next clinical implementation.

## Supplementary Information

**Additional file 1.**

## Data Availability

The data used to support the findings of this study are available from the corresponding authors upon request. Data use is subject to the approval of the Ethics Committee of the Renmi Hospital of Wuhan University.

## Abbreviations

TSR: Tumor-stroma ratio
ER: Estrogen receptor
PR: Progesterone receptor
HER-2: Human epidermal growth factor receptor-2
TMA: Tissue Microarray
H&E: Hematoxylin and eosin
5-DFS: 5-Year disease-free survival
TNBC: Triple-Negative Breast Cancer
HR: Hazard ratio
