# Supplementary material for: Tumour stroma ratio is a potential predictor for 5-year disease-free survival in breast cancer
Source: BMC Cancer. 2022 Oct 21;22:1082. doi: 10.1186/s12885-022-10183-5 (PMC9585868; doi:10.1186/s12885-022-10183-5)
Supplement: Supplementary file 1 — Additional file 1. [file 12885_2022_10183_MOESM1_ESM.doc]

sTable 1 Univariate and multivariate analysis of the HER2-positive breast cancer disease-free survival by Cox regression analysis

| Characteristics | Number of patients | Univariable analysis | | Multivariable analysis | |
| --- | --- | --- | --- | --- | --- |
|  |  | HR(95%CI) | P Value | HR(95%CI) | P Value |
| Age |  |  |  |  |  |
|  | 61 | 0.98(0.94-1.03) | 0.463 | 1.02(0.95-1.11) | 0.567 |
| Histological Grade |  |  |  |  |  |
| I | 3 | ref |  | ref |  |
| II | 38 | 1.66(0.48-4.19) | 0.914 | 1.92(0.30-5.10) | 0.912 |
| III | 20 | 2.95(0.36-6.87) | 0.897 | 2.59(0.41-5.97) | 0.899 |
| Nodal stage |  |  |  |  |  |
| Negative | 27 | ref |  | ref |  |
| Positive | 34 | 14.62(4.38-48.73) | 0.000 | 13.64(3.46-53.86) | 0.000 |
| Menopausal status |  |  |  |  |  |
| Premenopausal | 32 | ref |  | ref |  |
| Postmenopausal | 29 | 1.40(0.69-2.84) | 0.348 | 0.47(0.13-1.75) | 0.262 |
| Tumor size (in cm) |  |  |  |  |  |
| ≤2 | 5 | ref |  | ref |  |
| 2< T ≤5 | 38 | 2.99(0.40-22.40) | 0.287 | 1.58(0.19-13.06) | 0.672 |
| >5 | 18 | 5.09(0.66-39.27) | 0.118 | 2.01(0.24-16.70) | 0.520 |
| TSR |  |  |  |  |  |
| Stroma-low | 34 | ref |  | ref |  |
| Stroma-high | 27 | 2.76(1.35-5.67) | 0.006 | 4.23(1.73-10.32) | 0.002 |

sTable 2 Univariate and multivariate analysis of the TNBC disease-free survival by Cox regression analysis

| Characteristics | Number of patients | Univariable analysis | | Multivariable analysis | |
| --- | --- | --- | --- | --- | --- |
|  |  | HR(95%CI) | P Value | HR(95%CI) | P Value |
| Age |  |  |  |  |  |
|  | 63 | 1.02(0.98-1.06) | 0.375 | 0.99(0.93-1.05) | 0.700 |
| Histological Grade |  |  |  |  |  |
| I | 7 | ref |  | ref |  |
| II | 29 | 2.27(0.52-9.90) | 0.274 | 1.43(0.31-6.58) | 0.647 |
| III | 27 | 3.47(0.80-14.99) | 0.096 | 1.81(0.38-8.56) | 0.454 |
| Nodal stage |  |  |  |  |  |
| Negative | 28 | ref |  | ref |  |
| Positive | 35 | 2.91(1.39-6.06) | 0.004 | 3.14(1.45-6.82) | 0.004 |
| Menopausal status |  |  |  |  |  |
| Premenopausal | 30 | ref |  | ref |  |
| Postmenopausal | 33 | 1.53(0.79-2.98) | 0.209 | 1.76(0.60-5.20) | 0.303 |
| Tumor size (in cm) |  |  |  |  |  |
| ≤2 | 7 | ref |  | ref |  |
| 2< T ≤5 | 44 | 2.01(0.47-8.48) | 0.344 | 3.31(0.74-14.76） | 0.116 |
| >5 | 12 | 4.29(0.92-19.96) | 0.064 | 7.89(1.52-40.91) | 0.014 |
| TSR |  |  |  |  |  |
| Stroma-low | 37 | ref |  | ref |  |
| Stroma-high | 26 | 2.59(1.34-5.03) | 0.005 | 2.68(1.29-5.59) | 0.008 |

sTable 3 Univariate and multivariate analysis of the luminal–HER2-negative breast cancer disease-free survival by Cox regression analysis

| Characteristics | Number of patients | Univariable analysis | | Multivariable analysis | |
| --- | --- | --- | --- | --- | --- |
|  |  | HR(95%CI) | P Value | HR(95%CI) | P Value |
| Age |  |  |  |  |  |
|  | 116 | 0.97(0.93-1.01) | 0.197 | 0.98(0.91-1.06) | 0.683 |
| Histological Grade |  |  |  |  |  |
| I | 30 | ref |  | ref |  |
| II | 74 | 2.65(0.59-11.87) | 0.201 | 1.47(0.31-6.98) | 0.624 |
| III | 12 | 22.70(4.92-104.63) | 0.000 | 11.40(2.05-63.51) | 0.005 |
| Nodal stage |  |  |  |  |  |
| Negative | 54 | ref |  | ref |  |
| Positive | 62 | 5.16(1.76-15.10) | 0.003 | 3.28(1.04-10.35) | 0.042 |
| Menopausal status |  |  |  |  |  |
| Premenopausal | 72 | ref |  | ref |  |
| Postmenopausal | 44 | 0.92(0.40-2.10) | 0.841 | 1.47(0.34-6.29) | 0.604 |
| Tumor size (in cm) |  |  |  |  |  |
| ≤2 | 23 | ref |  | ref |  |
| 2< T ≤5 | 80 | 5.28(0.70-39.69) | 0.106 | 3.64(0.47-28.33) | 0.216 |
| >5 | 13 | 12.85(1.55-106.83) | 0.018 | 2.88(0.31-26.95) | 0.353 |
| TSR |  |  |  |  |  |
| Stroma-low | 82 | ref |  | ref |  |
| Stroma-high | 34 | 2.21(0.99-4.94) | 0.053 | 2.59(1.09-6.15) | 0.031 |
